# The human adrenal cortex: growth control and disorders

**DOI:** 10.6061/clinics/2018/e473s

**Published:** 2018-08-28

**Authors:** Claudimara Ferini Pacicco Lotfi, Jean Lucas Kremer, Barbara dos Santos Passaia, Isadora Pontes Cavalcante

**Affiliations:** Departamento de Anatomia, Instituto de Ciencias Biomedicas, Universidade de Sao Paulo, Sao Paulo, SP, BR

**Keywords:** Human Adrenal Cortex, Hyperplasia, Adrenocortical Tumors

## Abstract

This review summarizes key knowledge regarding the development, growth, and growth disorders of the adrenal cortex from a molecular perspective. The adrenal gland consists of two distinct regions: the cortex and the medulla. During embryological development and transition to the adult adrenal gland, the adrenal cortex acquires three different structural and functional zones. Significant progress has been made in understanding the signaling and molecules involved during adrenal cortex zonation. Equally significant is the knowledge obtained regarding the action of peptide factors involved in the maintenance of zonation of the adrenal cortex, such as peptides derived from proopiomelanocortin processing, adrenocorticotropin and N-terminal proopiomelanocortin. Findings regarding the development, maintenance and growth of the adrenal cortex and the molecular factors involved has improved the scientific understanding of disorders that affect adrenal cortex growth. Hypoplasia, hyperplasia and adrenocortical tumors, including adult and pediatric adrenocortical adenomas and carcinomas, are described together with findings regarding molecular and pathway alterations. Comprehensive genomic analyses of adrenocortical tumors have shown gene expression profiles associated with malignancy as well as methylation alterations and the involvement of miRNAs. These findings provide a new perspective on the diagnosis, therapeutic possibilities and prognosis of adrenocortical disorders.

## HUMAN FETAL DEVELOPMENT OF THE ADRENAL CORTEX

### Fetal zone formation

The human adrenal glands develop from two different embryological tissues. The medulla is derived from neural crest cells, while the cortex develops from the intermediary mesoderm. The earliest recognizable form of the adrenal gland occurs 28-30 days postconception (DPC) as the adrenogonadal primordium (AGP), which is detected by the expression of steroidogenic factor 1 (SF-1), also known as NR5A1 1. After migration in the dorsolateral direction, a subset of AGP cells expressing high levels of SF-1 forms the adrenal primordial (AP) or adrenal fetal zone (FZ). At 6 weeks of gestational age, the developing AP, located ventrolateral to the aorta, receives pheochromoblasts derived from neural crest cells that migrate through the AP cells and differentiate into the catecholamine-producing chromaffin cells of the adrenal medulla 2,3. By 50-52 DPC, the AP is encapsulated in a layer formed by fibroblast-like cells overlying the cortical zone. Meanwhile, the developing AP displays two zones, the inner FZ and the outer definitive zone (DZ). The FZ consists of large eosinophilic cells with characteristics typical of steroidogenic cells, whereas the DZ consists of small, compact basophilic cells arranged in clusters with the structural characteristics of cells in a proliferative state. As gestation advances, cells in the inner region of the DZ show lipid accumulation in the cytoplasm, resembling that of active steroidogenic cells with the capacity for cortisol production. At approximately the 30^th^ week of gestation, the fetal adrenal gland resembles a rudimentary adrenal gland with zona glomerulosa (ZG)-like and zona fasciculata (ZF)-like components 4.

## FACTORS INVOLVED IN THE REGULATION OF FETAL ADRENAL DEVELOPMENT

The factors involved in the regulatory mechanisms of fetal adrenal development include SF-1, DAX1, ACTH, CRH and CRH-homologous peptides, IGF1/2 and WT1. SF-1 is a crucial factor for the initial development and maturation of the adrenal cortex. SF-1-knockout mice 5 and some patients show adrenal aplasia in the absence of SF-1 expression 5,6, while overexpression of SF-1 in mice results in proliferation and neoplasia of the adrenal cortex 7. An investigation of the SF-1 gene revealed a mouse fetal adrenal-specific enhancer (FAdE) containing binding sites for the transcriptional complex Prep1-Hox9-b-Pbx1 and SF-1. The transcriptional complex Prep1-Hox9-b-Pbx1 initiates FAdE-mediated SF-1 expression, which subsequently regulates itself by maintaining FAdE-mediated SF-1 expression in the AP 8. FAdE is not necessary in mice after E14.5; however, no enhancer was characterized in the mouse DZ 9. Whether there is enhancer-mediated SF-1 activity during the FZ/DZ transition in the human adrenal gland is also not known.

The dosage-sensitive sex reversal-adrenal hypoplasia congenita critical region on the X chromosome, gene 1 (DAX1), also known as Nr0b1, is an orphan nuclear receptor. It interacts with SF-1 and LRH-1 (liver receptor homolog-1, NR5A2), and functional studies have shown that DAX-1 is a repressor of SF-1 and LRH-1 10,11. The biological role of Dax1 remains unclear. The deletion of exon 2 of Dax1 resulted in animals with normal adrenal function 12; however, adrenal insufficiency develops with time in aging Dax1-deleted mice 13. Dax1 is expressed in a population of stem cells from the adrenal cortex, and its presence represses differentiation, allowing the expansion of progenitor cells. Conversely, in the absence of Dax1 and the presence of adrenocorticotropic hormone (ACTH) stimulation, progenitor cells differentiate prematurely into steroidogenic cells 14. Moreover, Dax1 may serve to repress FAdE activity in the mouse adrenal gland during the FZ to DZ transition 9. In fact, the regulation of Dax1 expression maintains the balance between progenitor stem cell renewal and adrenocortical differentiation, which involves different factors such as SF-1, ACTH and Wnt signaling 15. Recently, studies *in vivo* and *in vitro* have defined a repressor function of SF-1 SUMOylation and Dax1 in the physiological interruption of FAdE-mediated SF-1 expression and the resultant regression of the mouse X-zone 16.

As the outer DZ emerges, ACTH participates in the regulation of steroidogenesis and the maintenance of the adrenal cortex. ACTH is essential for morphological and functional adrenal cortex development after the first trimester in human pregnancy 17. ACTH is a peptide hormone with 39 amino acids whose secretion is controlled by a 41-amino acid peptide, corticotropin-releasing hormone (CRH), in the anterior pituitary. In the adrenal cortex, ACTH binds to melanocortin receptor 2 (MC2R), activating cAMP-dependent protein kinase (PKA) and ERK/MAPK 18. CRH is produced in the hypothalamus and stimulates corticotroph cells in the anterior pituitary to produce a precursor polypeptide with 241 amino acid residues, proopiomelanocortin (POMC), which produces ACTH (among other peptides) after cleavage. CRH, the homologous peptides urocortin (UCN) 1, 2 and 3, and CRH receptors are found in the FZ and DZ 19 in distinct patterns during adrenal development, suggesting local regulation by CRH and related peptides 20. The role of ACTH in adrenal development is performed partially through the stimulation of growth factors such as insulin-like growth factor 1–2 (IGF1 and IGF2), both expressed in the adrenal gland 21. IGF2 is more highly expressed in the fetal adrenal gland, whereas IGF1 predominates in the adult adrenal gland. However, IGF2 is also present (although at lower levels than in the fetal adrenal gland) in progenitor cells localized in the outer adult adrenal zone, specifically in capsule and subcapsule cells 22. Mouse embryos lacking functional insulin/IGF signaling maintained the AGP in an undifferentiated state and presented complete agenesis of the adrenal cortex, failure in the testicular genetic program and delay in ovarian differentiation, demonstrating the crucial role of IGF signaling in different aspects of AGP development 23. Wilms tumor suppressor (WT1) is a fundamental factor in the AGP, as ectopic WT1 expression maintains the AGP in an undifferentiated state, preventing the differentiation of progenitor cells into steroidogenic cells. Moreover, through the control of WT1, GATA4, GLI1, and TCF21 expression in the mouse adrenal cortex, progenitor/stem populations can provide and maintain steroidogenic cells in the adrenal gland 24.

The extracellular matrix (ECM) could play an important role during human adrenal gland development 25. The identification of ECM components and their integrin receptors in the second-trimester human fetal adrenal gland revealed that collagen IV was distributed throughout the fetal gland. Laminin was predominant in the DZ, whereas fibronectin was predominant in the FZ. Additionally, the α2- and α3-subunits of integrin were detected at high levels throughout the entire adrenal cortex, but only the β3-integrin subunit was detected in chromaffin cells 26, suggesting a specific role during adrenal gland development. Indeed, experiments using human fetal adrenal gland cultures showed that the presence of collagen IV in the matrix substrate increased the effect of ACTH stimulation on the levels of dehydroepiandrosterone sulfate (DHEA-S) and cortisol in the culture medium. Moreover, the presence of fibronectin in the substrate enhanced the secretion of DHEA-S but impaired that of cortisol 26. In the DZ, laminin protected cells from apoptosis induced by angiotensin 2 (Ang II), which is consistent with the low level of cell death in the DZ 27. Moreover, the collagen and laminin expressed in the DZ can contribute to maintaining the high level of proliferation needed to ensure fetal adrenal growth 28. Taken together, these results provide evidence of the contribution of the extracellular microenvironment not only to plasticity but also to responsiveness to hormones in human fetal adrenal gland development.

## TRANSITION OF THE FETAL ZONE TO THE DEFINITIVE ZONE/ADULT ADRENAL CORTEX

Studies of the transition of the mouse FZ to the DZ and adult adrenal cortex provide insights into the mechanisms that regulate this transition. Zubair et al. 8,9,29 concluded that DZ cells are derived from FAdE-expressing cells of the FZ. Conversely, findings involving the activation of the sonic hedgehog (SHH) pathway and its effector Gli1, a marker of steroidogenic adrenal cell progenitors, provide evidence that Gli1-positive/SF-1-negative adrenal capsule cells give rise to the DZ 30.

Research by Wood et al. 31 integrates observations regarding the origin of adrenocortical adult steroidogenic cells from both the FZ and the adrenal capsule. These studies demonstrated that subsets of Gli-positive capsular cells are descended from cells of the FZ that previously expressed SF-1. Moreover, capsule cells expressing the transcription factor TCF21, a known inhibitor of SF-1 32,33, give rise to nonsteroidogenic stromal adrenocortical cells. These observations indicate the origin of the adult adrenal cortex from either fetal cortical cells or fetal cortex-derived capsular cells.

After birth, the FZ undergoes rapid involution by apoptosis 34, combined with differentiation of the DZ and development of the ZF and ZG, under the influence of the hypothalamus-pituitary-adrenal (HPA) axis (ACTH), Ang II and other trophic factors 35. As a consequence, the weight of the adrenal gland decreases by 50%, and regardless of gestational age at birth, gland size decreases to its normal childhood size within two weeks after birth 36. At approximately 6-9 years old, the zona reticularis (ZR) begins to form in the inner boundary of the ZF with the medulla, a process known as adrenarche. Adrenarche is characterized by the production of adrenal androgens and proliferation of the ZR 37,38, but the mechanism and control factors are not completely known. The postnatal mouse adrenal cortex contains a remnant of the ZF, the X-zone, located adjacent to the adrenal medulla 39. The X-zone disappears at puberty in males and during the first pregnancy in females 40. As the enzyme cytochrome P450 17A1 (Cyp17A1), which is required for steroidogenic hormones, is absent in the adrenals of postnatal mice, the mouse adrenal does not produce androgens 41.

## HUMAN ADULT ADRENAL CORTEX

### The structure of the human adrenal cortex

The adult adrenal gland is composed of two parts, the medulla and the cortex. The human adult adrenal gland consists of three compartments with distinct morphological and functional characteristics called the zona glomerulosa (ZG), zona fasciculata (ZF) and zona reticularis (ZR). The ZG is the outermost and most compact compartment composed of ovoid cells forming rosettes similar to glomeruli. These cells, which are also called aldosterone-producing cell clusters (APCCs), express aldosterone synthase (CYP11B2), primarily in the outer layers 42. Under the ZG lies the ZF, which is composed of large cells in radial cords that form fascicles and constitute the major part of the gland. The inner adrenal cortex zone bordering the medulla is the ZR, which is composed of cords of cells scattered in different directions to form a network.

The adrenal glands are highly vascular and are supplied by three arteries, the superior, middle and inferior adrenal arteries, which are ramifications of the phrenic, aortic and renal arteries, respectively. The adrenal arteries supply the adrenal glands with approximately 50 arterioles, forming a capsular arteriolar plexus. This arteriolar plexus projects cord-like sinusoid capillaries deeper into the cortex. In the ZR, another sinusoid plexus is formed that empties into a central vein in the medulla. The right adrenal vein drains directly to the vena cava, while the longer left adrenal vein drains into the left renal vein. The rich innervation of the adrenal glands comes from the celiac plexus and the abdominopelvic splanchnic nerves of the sympathetic autonomic nervous system in conjunction with parasympathetic bundles from the phrenic and vagal nerves. After penetrating the adrenal capsule, they form a subcapsular nerve plexus whose fibers extend to both the cortex and medulla surrounding the cells of these regions. The intrinsic innervation of the adrenal gland arises from ganglion cells distributed in the subcapsular region, cortex and medulla 43. In the adrenal medulla alone, the preganglionic sympathetic fibers cross the paravertebral and prevertebral ganglia without synapsing to terminate on the postganglionic neurons and chromaffin cells 44.

## THE FUNCTION OF THE HUMAN ADRENAL CORTEX

The function of the adrenal gland is steroid hormone production. Three main types of hormones are produced: glucocorticoids (cortisol, corticosterone), mineralocorticoids (aldosterone, deoxycorticosterone) and androgens (sex steroids). Cholesterol is the precursor of all adrenal steroid hormones 45; its principal source is low-density lipoprotein (LDL) cholesterol uptake by LDL receptors present in adrenal cells 46. LDL-LDLR is internalized by endocytosis, and the vesicles formed fuse with lysozymes, where free cholesterol is produced after hydrolysis. Cholesterol can be generated de novo from acetyl coenzyme A, and HDL cholesterol can be utilized through the scavenger receptor class B type 1 (SR-B1) 47.

## MINERALOCORTICOID SECRETION: THE RENIN-ANGIOTENSIN-ALDOSTERONE AXIS

Aldosterone is the primary mineralocorticoid produced by ZG cells under the principal control of Ang II, potassium and, to a lesser extent, ACTH. In contrast, somatostatin, heparin, atrial natriuretic factor, and dopamine inhibit Ang II synthesis. Angiotensinogen is synthesized in the liver and converted to angiotensin I (Ang I) by renin in the kidney. Ang I is later converted to Ang II by angiotensin converting enzyme (ACE) in the lung. The secretion of aldosterone is restricted to glomerulosa cells due to the specific presence of the enzyme aldosterone synthase, CYP11B2 48. Ang II and potassium stimulate aldosterone secretion by increasing the transcription of CYP11B2, but ACTH has no effect on CYP11B2 gene or enzyme activity. ACTH increases aldosterone by stimulating the early steps of the steroidogenesis pathway, contributing to increased aldosterone. After binding to nuclear mineralocorticoid receptors, aldosterone causes increased reabsorption of sodium and excretion of both potassium and hydrogen ions in kidney tubules. Other aldosterone target tissues are the colon and salivary glands.

## GLUCOCORTICOID SECRETION: THE HYPOTHALAMUS-PITUITARY-ADRENAL (HPA) AXIS

In humans, cortisol is the principal glucocorticoid produced by the human ZF under the control of ACTH, increasing blood glucose concentration through its action on glycogen, protein and lipid metabolism. Glucocorticoids inhibit glucose uptake in muscles, causing insulin resistance in muscle tissue, and lipolysis in adipose tissue, resulting in the release of fatty acids into circulation 49. ACTH is synthesized by the anterior pituitary as part of a 241-amino acid precursor, POMC. ACTH is secreted by the anterior pituitary under the influence of CRH, arginine vasopressin (AVP) and some cytokines 50. CRH secretion from the hypothalamus is regulated by circadian rhythms and by stressors acting on the hypothalamus, such as hypoglycemia, hypotension and fever. The secretion of both CRH and ACTH is inhibited by glucocorticoids in negative feedback control of the HPA axis.

## ADRENAL ANDROGEN SECRETION

ZR cells in the human adrenal produce the precursor androgens dehydroepiandrosterone (DHEA), dehydroepiandrosterone sulfate (DHEA-S), androstenedione androstenediol and 11β-hydroxyandrostenedione (11OHA) 51. These androgens have weak activity, but they are precursors for peripheral tissue conversion to testosterone and estrogens such as estradiol 52. These androgens and precursor steroids produced in the human adrenal seem to be stimulated by ACTH 53. In addition, there is evidence that luteinizing hormone and chorionic gonadotropin (LH/hCG) could regulate androgen synthesis independently of ACTH 54. However, the mechanism that regulates the synthesis of adrenal androgens has not yet been fully characterized.

## MAINTENANCE OF THE ADRENAL CORTEX

### Sonic hedgehog and Wnt/β-catenin pathways

Several findings have suggested that cells proliferate in the outer region of the adrenal cortex (capsule, subcapsule and outer ZG cells) and migrate to the inner regions during differentiation (inner ZG and ZF cells), while senescence occurs at the boundary between the cortex and medulla (ZR cells) 55-57.

Two pathways are involved in mouse adrenal gland development and maintenance: the Sonic hedgehog (SHH) and Wnt/β-catenin pathways. In studies utilizing lineage tracing in mouse embryos, SHH was found in progenitor cells residing in the subcapsular ZG 30. In adult adrenal glands, SHH-positive cells colocalize with SF-1 in nonsteroidogenic cortical cells of the ZG but not in differentiated ZG or ZF cells. Both ZG and ZF cells express SF-1; in addition, ZG and ZF cells express Cyp11b2 and Cyp11b, respectively. All of these molecules are markers of fully differentiated steroidogenic cells. Other findings involving the loss of SHH in the adrenal cortex have shown reduced proliferation of capsular cells and decreases of 50-70% in the adrenal size of SF-1-Cre mice, who remain smaller as adults 58. However, the remaining adrenal cortex maintains its steroidogenic function, indicating that SHH is not related to the differentiation of the adrenal cortex. These results implicate the SHH pathway in the maintenance of the fetal and adult adrenal cortex. In a recently created mouse model, the transcriptional regulators YAP (Yes-associated protein) and TAZ (transcriptional coactivator with PDZ-binding motif), both effectors of the Hippo signaling pathway, were conditionally deleted in steroidogenic cells 59. Male mice showed downregulation of SHH, age-dependent degeneration of the adrenal cortex, an increase in apoptosis and a reduction in steroidogenic gene expression. In contrast, no gross degenerative changes were observed in the adrenal glands of females, although steroidogenic capacity and SHH expression were reduced, suggesting an important role for YAP and TAZ in the maintenance of the postnatal adrenal cortex.

To investigate the role of the Wnt/β-catenin pathway in the maintenance of the adrenal cortex, mouse Sf1/Cre transgenes were used to inactivate conditional β-catenin alleles 60. The analysis of fetal adrenal development after the complete inactivation of β-catenin showed decreased proliferation in presumed progenitor cells of the adrenal cortex and adrenal aplasia. In animals with lesser degrees of β-catenin inactivation, age-dependent degeneration of the adrenal cortex occurred, demonstrating the role of the Wnt/β-catenin pathway in maintaining the fetal and adult adrenal cortex. In contrast, overactivation of β-catenin produced an increase in progenitor-like cells and tumor formation. Indeed, the Wnt pathway is frequently and aberrantly activated in adrenocortical tumors 61,62.

## EFFECTS OF ACTH AND N-POMC ON ADRENAL MAINTENANCE AND PROLIFERATION

The elimination of POMC products was shown to prevent development of the adrenal gland. Yaswen et al. 63, using mice lacking POMC-derived peptides (i.e., POMC-null mutant mice), showed defective adrenal development and undetectable plasma steroids. In another study using POMC -/- mice 64, adrenal glands were found in all mice, although they had reduced weight and disrupted cortical architecture compared with those of wild-type animals. Additionally, plasma corticosterone was undetectable, while aldosterone was significantly reduced. In POMC -/- mice treated for ten days with ACTH, the adrenal weight, adrenal morphology and plasma corticosterone levels were restored.

Estivariz et al. 65,66 tested peptides derived from pro-gamma-MSH, a smaller N-POMC peptide lacking gamma-MSH, which proved to be potent mitogens both *in vivo* and *in vitro*. One of them, N-POMC 1–28, was shown to have this activity. N-POMC 1–28 was isolated from human pituitary glands, but it was initially considered an artifact because it was not found in circulation 67. Since then, it has been tested and shown to be mitogenic in the murine adrenal gland. The first 28 amino acids of the N-terminal portion of POMC have been shown to be essential to the mitogenic activity of N-POMC 1–28 *in vivo* 68-70 and *in vitro* 71-73. Moreover, N-POMC 1–28 peptides prevent atrophy in the regenerating adrenal gland after hypophysectomy in rats by preventing the apoptosis of adrenocortical cells 69.

N-POMC 1–49 is an endogenous peptide produced after the cleavage of pro-gamma-MSH in the intermediary lobe of the pituitary. *In vitro* studies demonstrated that this peptide is mitogenic 71,73; however, *in vivo*, it does not induce enlargement of the adrenal gland 74. Previous research indicates that O-linked glycan must be present for the N-POMC 1–49 proliferative effect 75. However, this issue requires further investigation to understand the involvement of endogenous N-POMC 1–49 in adrenal proliferation and maintenance.

Although dependence on ACTH and N-POMC peptides is evident, and these peptides are crucial for maintenance of the adrenal cortex, other tissue components such as the vasculature 76, and factors such as neurotransmitters 77 may also have specific roles in this function. There is also the possibility of the integration of medullary, neural and vascular regulation of cortical function and maintenance as a consequence of the intimate relationship between these adrenal gland components.

## DISORDERS OF ADRENAL CORTEX GROWTH

### Adrenal hypoplasia

Adrenal hypoplasia is defined by underdevelopment or hypotrophy of the adrenal cortex as a consequence of distinct clinical conditions. Adrenal hypoplasia can be divided into two categories of disease: primary and secondary hypoplasia. Primary hypoplasia is characterized by hypofunctional and hypotrophic adrenals due to deficiencies observed in the formation and differentiation of the adrenal gland; this condition is called congenital adrenocortical hypoplasia. Four forms of congenital adrenal hypoplasia have been identified, as follows: 1) an X-linked form is caused by a mutation or deletion of the *DAX1* gene on the X chromosome 78; 2) the autosomal recessive form is due to a mutation or deletion of the SF-1 gene on chromosome 9q33 79; 3) a rare autosomal form includes IMAGE syndrome 80 and SERKAL syndrome 81, caused by mutations in *CDKN1C* and *WNT4*, respectively; and 4) ACTH resistance syndrome and familial isolated glucocorticoid deficiency syndromes are autosomal recessive diseases caused by mutations in genes related to ACTH function and signaling, such as MC2R and MRAP 82.

Mutations in two other genes have also been associated with ACTH resistance syndrome, the nicotinamide nucleotide transhydrogenase (NNT) gene 83 and the minichromosome maintenance-deficient 4 (MCM4) gene 84. The secondary form of adrenal hypoplasia is caused by pituitary or hypothalamic dysfunction, such as developmental abnormalities of the central nervous system, diseases of pituitary development, isolated ACTH deficiency, defects in POMC processing and enzyme convertase 1 (PCSK1), and nongenetic causes 85. These dysfunctions result in deficient ACTH synthesis and secretion, which culminates in adrenal hypofunction and hypotrophy due to a lack of adrenal stimulus.

## ADRENOCORTICAL HYPERPLASIA

### Congenital adrenal hyperplasia (CAH)

CAH is an inherited enzymatic deficiency in cortisol synthesis. The deficiency in cortisol production results in excessive secretion of ACTH, which in turn cannot block cortisol synthesis, leading to enlargement of the adrenal gland. The more severe forms (i.e., complete enzymatic defect) are called “classic” CAH, while the milder forms (partial enzymatic defect) are referred to as “nonclassic” CAH. All CAH forms are autosomal recessive disorders.

Deficiency in 21-hydroxylase (CYP21A2) is the most common cause of CAH, accounting for more than 90% of cases 86. A complete absence of CYP21A2 activity results in glucocorticoid and mineralocorticoid deficiencies as well as androgen excess (virilization). Nonclassic CYP21A2 deficiency does not produce adrenal insufficiency but is associated with premature puberty, hirsutism, acne, and irregular menses due to excess androgen. The gene encoding human CYP21A2 is located on chromosome 6p21.3 inside the human leukocyte antigen (HLA) major histocompatibility complex gene 87. Complete deletions, large gene conversions, and nonsense or frameshift mutations result in compromised CYP21A2 and the classic form of CYP21A2 deficiency. In nonclassic CYP21A2 deficiency, the alleles preserve some enzyme activity as well as cortisol and aldosterone production 88.

The other form of CAH is 11β-hydroxylase (CYP11B1) deficiency, which represents approximately 8% of all CAH cases 89. Patients with this form of CAH also exhibit decreased cortisol synthesis and adrenal androgen overproduction. In contrast to CYP21A2, patients with CYP11B1 deficiency present with hypertension and sometimes hypokalemia. The CYP11B1 gene is located on chromosome 8q21-22, approximately 40 kb from the homologous CYP11B2, the aldosterone synthase gene 90. More than 80 mutations have been described, but the relationship between genotype and phenotype remains unclear 91.

3β-Hydroxysteroid dehydrogenase type 2 (3βHSD2) deficiency is characterized by both mineralocorticoid and glucocorticoid deficiency as well as dehydroepiandrosterone (DHEA) production. *HSD3B2* is expressed exclusively in the adrenals and gonads, while HSD3B1, the homologous type I gene, is expressed in the placenta and peripheral tissues (skin, breast and prostate) 92. The *HSD3B2* gene is located on chromosome 1p13.1 and consists of four exons. Exons 2–4 are translated into a protein of 371 amino acids 93. Mutations (nonsense and frameshift) eliminate enzyme transcription and function, resulting in salt-wasting forms of 3βHSD2 deficiency, while single amino acid substitution decreases the affinity of the enzyme for substrate, leading to non-salt-wasting forms of 3βHSD2 deficiency 94.

Deficiency in 17α-hydroxylase (CYP17A1) is rare and results in no production of either cortisol or androgens, but the progesterone and aldosterone pathways are not affected. *CYP17A1* is encoded by a gene located on chromosome 10q24.3 that contains 8 exons encoding a 508 amino acid protein 95. More than 90 mutations have been described in countries where CYP17A1 is more prevalent, and specific mutations are present due to founder effects 96.

The most severe defect in steroidogenesis is lipoid congenital adrenal hyperplasia (LCAH), in which the patients' adrenal glands are greatly enlarged and replete with lipids. This type of hyperplasia is caused by a defect in the steroidogenic acute regulatory protein (StAR), which prevents the mobilization of cholesterol into the mitochondria, resulting in nonsteroid synthesis. P450-oxidoreductase deficiency combines features of CYP21A2, CYP17A1 and CYP19A1, which are associated with different degrees of severity. These patients have different skeletal malformations that are characteristic of Antley-Bixler syndrome 97.

Treatments for CAH include glucocorticoid and mineralocorticoid therapy as well as some experimental therapies other than glucocorticoids. However, treatment remains a major clinical challenge, and no consensus exists among practitioners 91.

## PRIMARY MACRONODULAR ADRENAL HYPERPLASIA (PMAH)

Described for the first time in 1964 98, PMAH is a rare cause of Cushing syndrome (CS), accounting for less than 2% of cases 99. In general, PMAH presents with bilateral macronodules and adrenal enlargement. The macronodules present with aberrant expression of different ectopic G-protein coupled receptors, such as gastric inhibitory polypeptide receptor, luteinizing hormone receptor and serotonin receptors 99,100. The binding of these aberrant receptors to their ligands mimics the result of ACTH binding to MC2R, activating the PKA pathway and increasing cortisol production 101. Additionally, the presence of ectopic POMC and ACTH in a subpopulation of cells within the hyperplastic nodules was described recently in several cases of PMAH in both tissue 102 and human cell cultures obtained from PMAH nodules 103. The production of ectopic ACTH in the nodules is at least partially regulated by the PKA pathway and may act in autocrine and paracrine manners 103, allowing a certain independence of cortisol synthesis in PMAH cells.

The molecular causes of PMAH have not been completely established, but several studies have tried to find a common event leading to the formation of macronodules and variable cortisol secretion. For example, mutations in the *GNAS* and *MC2R* genes have been reported, but they are present in only a limited number of cases. Recently, frequent germline mutations in the *ARMC5* gene have been reported in different potentially sporadic and familial cohorts (24-55%), making such mutations the most frequent molecular abnormality related to PMAH 104-108. The *ARMC5* gene is located on chromosome 16p11.2, and because germline mutations are associated with nodule-specific somatic mutations, this gene is hypothesized to be a tumor suppressor gene 104.

**Figure 1:**
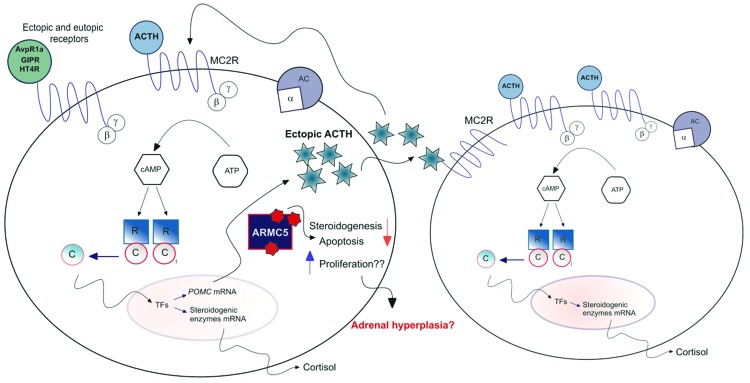
Steroidogenesis in adrenocortical cells is regulated by the binding of ACTH to MC2R, activating PKA pathway through the release of the catalytic subunit (C) by the action of cAMP in specific domains of the PKA, followed by the phosphorylation of transcription factors (TFs) in the nucleus that will increase the transcription of steroidogenic enzymes, leading to cortisol production. In addition to this classic signaling, PMAH cells can be abnormally regulated by aberrant receptors that mimic the action of ACTH when activating PKA; additionally, the activation of PKA regulates the production of ectopic ACTH within the hyperplastic nodules that will once again activate PKA independently of pituitary ACTH, allowing a certain independency to cell steroidogenesis. Additionally, mutations in ARMC5 are the main molecular events associated with PBMAH; ARMC5 decreases steroidogeneis, induces cell apoptosis and might also be involved in cell proliferation by mechanisms so far unknown.

Although little is currently known about the function of ARMC5, its importance in regulating steroidogenesis, proliferation and apoptosis has been described in cell lines and in PMAH cell cultures 103,104. Additionally, ARMC5 is important for the embryological development of mice, T cell differentiation, and immune response 109,110. Finally, ARMC5 has no enzymatic activity, and its function depends on interactions with other proteins 109. This interactivity may explain why it is involved in multiple distinct cellular mechanisms, as shown in Figure 1.

## ADRENAL TUMORS

Adrenocortical tumors (ACTs) are frequent neoplasms affecting 6-7% of the population 111. Most ACTs are benign, unilateral and nonfunctioning masses incidentally found during abdominal imaging in approximately 2% of the general population; they are designated incidentaloma. When functional, benign tumors often cause Cushing syndrome (CS), which originates from chronic exposure to variable amounts of glucocorticoids, mainly cortisol 112.

## ADRENOCORTICAL ADENOMAS (ACA)

Cortisol-producing adenomas are characterized by abnormally high levels of cAMP/protein kinase A (PKA) pathway activation. PKA is the main regulator of cortisol production and proliferation in adrenocortical cells 113. PKA is composed of four subunits, two catalytic and two regulatory, which are bound together under normal conditions. When cells are stimulated by the binding of ACTH to MC2R, a G-protein coupled receptor (GPCR), cAMP binds to the regulatory subunits and induces release of the catalytic subunits, which phosphorylate transcription factors in the nucleus, culminating in the transcription of steroidogenic enzymes and the synthesis of cortisol 114. Mutations in the gene encoding the alpha catalytic subunit of protein kinase A (*PRKACA*) have been reported in 30-65% of adenomas causing overt CS 115,116. The most frequent mutation described in this gene, p.Leu206Arg, leads to the replacement of a leucine residue at position 206 by arginine 117. These mutations constitutively activate the cAMP pathway, resulting in abnormal PKA activity, increased cortisol production, and tumor development. According to Lacroix and coworkers 118, the absence of mutations in the *PRKACA* gene in cases of low-cortisol-producing adenomas may explain why they rarely lead to CS over time 112.

Adrenocortical adenomas have also been associated with postzygotic mutations in the gene encoding the α subunit of the stimulatory guanine nucleotide-binding protein (*GNAS*). These mutations are normally associated with McCune-Albright syndrome (MAS), where mosaic gain-of-function *GNAS* mutations in patients with MAS lead to the constitutive activation of adenylyl cyclase and to clinical features such as fibrous dysplasia and cafe-au-lait skin pigmentation 119.

Although the mechanisms leading to the development of adrenocortical adenomas have not yet been completely identified, mutations in the *PRKACA* gene are considered the primary genetic alterations involved in the formation of these tumors through the cAMP/PKA signaling pathway, stimulating both cortisol secretion and cell proliferation.

Similar to adrenocortical adenomas, aldosterone-producing adenomas (APAs) are benign functional adrenocortical lesions. The mutations found in APAs are mostly related to the regulation of calcium and potassium channels, such as the somatic mutations found in *ATPA1A,* a gene encoding the alpha subunit of the sodium/potassium ATPase, and *ATP2B3,* a gene encoding the plasma membrane calcium-transporting ATPase3 120. Additionally, mutations in *KCNJ5,* which encodes potassium channels, were found in both sporadic and familial APA cases 121-123. These mutations damage the channel's permeability, allowing the entrance of sodium—instead of potassium—into the cell, resulting in depolarization.

## ADULT ADRENOCORTICAL CARCINOMAS

Adult adrenocortical carcinomas (ACCs) are rare tumors, with an incidence of 1-2 cases per million, that primarily affect adults over 40 years and are more frequent in women 124-126. ACCs may be associated with hereditary disorders such as Li-Fraumeni syndrome, multiple endocrine neoplasia type 1 (*MEN1*) and Lynch syndrome, which has a prevalence between 1-7% in patients with ACCs 127-129. ACCs are differentiated from ACAs by the presence of 3 or more Weiss criteria, including nuclear grade, mitotic rate, the presence of atypical mitoses, <25% clear cells, necrosis, >33% diffuse architecture, and vascular, sinusoid and capsule invasion 130,131. ACC can produce aldosterone, cortisol, androgens or estrogens, as described in Table 1 132,133. Alterations in the steroidogenic profile can be identified by urine tests for the diagnosis and follow-up of patients 129,134-136. The probability of the recurrence of ACC decreases when there is complete surgical resection of the adrenocortical mass 137,138. An adjuvant treatment for patients with ACC is the administration of mitotane (dichlorodiphenyldichloroethane, o,p'-DDD), whose accepted levels range from 14-20 mg/dl and whose common tolerated dose is 3-4 g per day 126.

There are well-established molecular markers of great clinical importance for adrenocortical tumors. Insulin-like growth factor (IGF2) expression is associated with a malignant ACC phenotype 139. In fact, it was shown *in vitro* that IGF2 induces proliferation, but its overexpression does not modify the phenotypic and molecular features of ACC cells 140. In contrast, a study that assessed IGF2 protein expression and overall survival (OS) found that high IGF2 expression is associated with longer OS, suggesting that IGF2 is not a prognostic factor for ACC progression or metastasis 141. Only in adult ACTs were differences in *IGF2* expression observed between ACA and ACC, while in pediatric ACTs, the *IGF1R* gene was overexpressed in carcinomas 142. Inhibition of the IGF signaling pathway has been proposed as a promising treatment 142, and in combination with the administration of mitotane, it reduces tumor growth 143. Moreover, the methylation levels of the *IGF2* promoter region may be a marker for distinguishing different types of ACTs 144.

Alterations in the Wnt/β-catenin signaling pathway are associated with gastric and ovarian cancer, gastrointestinal stromal and myeloid tumors and teratocarcinosarcomas 145-149. The anomalous expression of β-catenin protein, characterized by its accumulation in the cytoplasm and/or displacement to the nucleus, is a common finding in ACTs 150. This abnormality occurs in 77% of ACC cases and 38% of ACA cases; however, the expression of β-catenin is diffuse only in ACC 150. Other studies have found no prevalence of β-catenin protein or gene expression in ACC or ACA 151-152. In addition, β-catenin expression is not useful for predicting malignancy in ACCs 151. However, inhibition of the Wnt/β-catenin signaling pathway reduces cell viability and could be a treatment option for ACC patients 153.

ZNRF3 (zinc and ring finger 3) is an E3 ubiquitin ligase capable of negatively regulating the Wnt/β-catenin signaling pathway 154. The *ZNRF3* gene is amplified and exhibits homozygous deletions and mutations in ACCs 155,156. In a global study, the *ZNRF3* gene displayed the most frequent genetic alterations, suggesting that ZNRF3 is a tumor suppressor in ACCs 155.

In comprehensive molecular studies, *TP53* was the gene with the highest rate of genetic alterations in the analyzed groups 156,157. The R337H germline mutation in the *TP53* gene is an important molecular marker in pediatric tumors. It is present in only 13.5% of adult patients with ACTs; however, such mutations are associated with poor prognosis 158.

ACCs usually exhibit high expression of Ki67 protein, a marker of proliferating cells 159,160 that has been analyzed together with histopathological markers 161. Using a large group of patients with ACC, Ki67 was identified as the factor that most accurately d recurrence in patients who underwent complete surgical resection 162. Moreover, Ki67 is considered a predictor of disease-free time and OS 160.

Despite being involved in steroidogenesis, SF-1 exhibits no differential expression between functioning and nonfunctioning adrenocortical tumors 163. *SF-1* copy number and SF-1 protein expression are not determining factors in carcinomas, with increased expression in 10% and 19% of cases, respectively 163. SF-1 is regulated by dosage-sensitive sex reversal-adrenal hypoplasia congenita critical region on the X chromosome, gene 1 (DAX-1) and by upstream stimuli 1 and 2 (USF-1 and USF-2) 10,164. Another factor that regulates SF-1 is transcription factor 21 (TCF21/POD1), which binds to the proximal E-box region of SF-1 and negatively regulates the expression of both SF-1 and the cholesterol-transporting protein StAR 32,33,165. In adult ACTs, *TCF21* is expressed at a lower level in ACC than in ACA or normal adrenocortical tissue 33,166. In ACC, *TCF21* expression is negatively correlated with the cell cycle, mRNA surveillance and glycosaminoglycan biosynthesis-heparan sulfate pathways. The cell cycle parameter was the most significant, including effects on the genes *BUB1B* and *CDK1* 33.

Recent wide-ranging molecular studies have shown new genetic alterations that define a distinct profile of ACCs 155,157. An analysis of 91 ACC cases identified mutated genes as well as loss of heterozygosity (LOH) and increased copy number, which are common genetic alterations in ACCs. Key genes found altered in ACCs included TP53, β-catenin (CTNNB1) and telomerase reverse transcriptase (TERT) 157. These recently highlighted genetic alterations are summarized in Table 2.

Carcinomas have significantly different methylation levels from metastasis masses, adenomas and normal tissues; in general, carcinomas are hypomethylated compared to adenomas 167. Methylation levels and their association with gene downregulation have been investigated as predictors of prognosis in adult ACCs 168. In a comprehensive study, 212 CpG islands were found to be hypermethylated in ACCs compared to ACAs. Moreover, upon demethylation treatment in H295R adrenocortical carcinoma cells, the hypermethylated genes showed increased gene expression 169.

Small noncoding RNA molecules (miRs) modulate the expression of target genes by degrading mRNA posttranscriptionally or by inhibiting translation 170. Two separate studies have shown the potential of miRs as potential biomarkers in ACC, and miR profiles have been characterized in ACC 171,172. miR-195 and miR-483-5p (which map to the second intron of the *IGF2* gene) have been shown to have predictive value for outcomes in ACC patients 173,174. Another group found that miR-483-5p and miR-34a are candidate serum biomarkers for adrenocortical tumors, with 74% and 81% accuracy, respectively 175. More recently, a quantitative real-time assay was developed to measure miR483 and miR483-5p levels (as a liquid biopsy component) in plasma samples from 27 patients with ACC 176. The miR483-5p level was able to predict recurrence but not OS. In addition, miR483 and miR483-5p levels correlated with the number of circulating tumor cells (CTCs) detected in the same blood samples. In another study, the miRNA profile of ACTs showed 95% accuracy for discrimination between ACC and ACA 177. An miRNA profile of adrenocortical tumors based on multiple studies is presented in Table 3 173-175,177-180.

Long noncoding RNAs (lncRNAs) in adrenocortical tumors display tissue-specific expression and may be involved in transcriptional regulation and silencing 181,182. The lncRNA profile showed differential expression of 85 lncRNAs between carcinomas and adenomas. In comparison with normal adrenal tissue, 956 lncRNAs were differentially expressed in ACC, including GAS5, H19, MALAT1 and PRINS 183.

## PEDIATRIC ADRENOCORTICAL TUMORS

A dramatic remodeling of the adrenal cortex structure takes place after birth, with massive apoptosis of the FZ and, simultaneously, a progressive differentiation into distinct zones of adult adrenal cortex. Therefore, adrenocortical pediatric malignancies may be considered a disturbance of the normal development process. Pediatric adrenocortical tumors are a rare malignity, with a worldwide incidence estimated at 0.3/million/year, and are more frequent in girls than in boys. The incidence is higher in children under 5 years of age and above 10 years of age, and they exhibit virilization symptoms that may be associated with CS. The Weiss score system for adult ACTs is well established 184; however, in children, the ACT pathological criteria for malignancy remain uncertain 185. Certain criteria are prognostically favorable, such as a tumor grade of stage I at diagnosis, patient age under 4 years, tumor weight ≤200 g, volume <200 cm^3^, and symptoms limited to virilization only 186.

Pediatric adrenocortical tumors are more frequently associated with Beckwith-Wiedemann syndrome 187,188 and Li-Fraumeni syndrome (LFS) 189,190. Beckwith-Wiedemann syndrome causes adrenocortical hyperplasia and various neoplasms with variable malignancy. It is caused by genetic defects such as uniparental disomy in the 11p15 chromosomal region 191, whose consequence is the overexpression of IGF2.

LFS is a familial cancer predisposition disorder caused by germline mutations in the tumor suppressor gene TP53. LOH on chromosomes 11 and 17 is the hallmark of pediatric adrenocortical tumors associated with germline TP53 mutations 192. However, approximately 50% of pediatric ACTs do not harbor TP53 mutations. Recently, Pinto et al. 192 showed that CTNNB1 mutations occurred almost exclusively in pediatric ACTs without germline TP53 mutations. The high incidence of childhood ACT in LFS suggests that the absence of TP53 may lead to increased proliferative capability, promoting further mutations, clonal expansion and tumorigenesis.

In southern Brazil, the incidence of TP53 germline mutations is estimated to be between 3.4 and 4.2 per million children 193. Ribeiro et al. 194 demonstrated that a TP53-specific mutation, p.R337H, was related to a high prevalence of these tumors. A founder effect is responsible for the dissemination of the TP53/p.R337H mutation in Brazil 195.

Although pediatric adrenocortical tumors are highly associated with germline TP53 mutations, only approximately 4% of these cases develop tumors, suggesting other genetic alterations. A genomic study 192 showed that select cases with an R337H mutation presented with secondary genetic events, such as initial LOH on chromosomes 11 and 17p, followed by dysregulation of *IGF2* expression and the acquisition of *ATRX* (a DNA helicase) mutations 192. Another finding is LOH of the 11p15 region, leading to IGF2 overexpression from the paternal allele. Transgenic mouse models demonstrate that IGF2 overexpression is not sufficient to promote adrenocortical tumors 196; such promotion occurs only in cooperation with β-catenin 197.

The most common alteration found in pediatric adrenocortical tumors was the amplification and gain of chromosome 9q34 close to where the NR5A1/SF-1 gene is situated 198. In pediatric ACTs, the SF-1 gene is amplified, and the SF-1 protein is overexpressed, but this alteration seems to be unrelated to malignancy 199,200.

Strongly downregulated genes in pediatric ACT include 3-β-hydroxysteroid dehydrogenase (HSD3B2) 201, a steroidogenic enzyme expressed in the ZG and ZR, which is involved in aldosterone and cortisol synthesis. This finding reinforces the hypothesis of disturbances in fetal differentiation. Another downregulated gene is NOV/CCN3, which encodes a protein with proapoptotic function in adrenocortical cancer cells, suggesting that a deficient regression process in the FZ may be part of tumor formation 202.

A set of miRNAs that are differentially regulated in pediatric ACT has been identified, including miR-99a and miR-100, which are downregulated. Functional analysis of these miRNAs has shown that they are able to downregulate expression of the insulin-like growth factor-mammalian target of rapamycin (mTOR)-raptor signaling pathway 203. This pathway is upregulated in childhood ACT, and its inhibition decreases adrenocortical cell proliferation 204,205.

Pediatric adrenocortical tumors differ from adult ACT in their origin, molecular alteration and evolution. The identification of molecular and pathway markers is still a challenge; however, genomic analyses of genetic alterations and gene expression profiles provide important new insights into the pathogenesis and molecular classification of pediatric ACT 192.

Adrenocortical disorders result from misregulation of the adrenal cortex, generally leading to abnormal growth of the adrenal glands. However, each abnormality described in this review has its own unique molecular basis, which makes the diagnosis and treatment of patients challenging. The use of whole-genome sequencing to identify genes that can regulate the appearance or even the severity of adrenocortical diseases has led to a number of discoveries, especially related to ACC, ACA and PMAH. The recent description of several molecular markers in ACCs and the ARMC5 gene in large cohorts of patients diagnosed with PMAH has allowed researchers to identify the pathways involved in the development of these tumors. In the future, these findings may make it possible to target molecular alterations in a subset of patients with particular molecular profiles and thus control the abnormal growth of these adrenal masses.

## AUTHOR CONTRIBUTIONS

Lotfi CFP conceived and designed the study and was responsible for data acquisition. Kremer JL, Passaia BS and Cavalcante IP were also responsible for data acquisition. Lotfi CFP was responsible for the manuscript drafting, critical revision of the manuscript and approval of the final version of the manuscript. All authors read and approved the final version of the manuscript.

## Figures and Tables

**Table 1:** Endocrine features of functional ACCs.

Secretion	Incidence	Symptoms	References
Aldosterone	rare	Hypertension, heart disease	Vilela and Almeida (132), Akerstrom et al. (133)
Cortisol	50%-80%	Osteoporosis, diabetes mellitus, muscle weakness, plethora	Else et al. (129)
Androgen	40%-60%	Increased libido, acne, male baldness, virilization, hirsutism, and menstrual abnormalities	Young (134), Else et al. (129)
Estrogen	1%-3%	Gynecomastia, testicular atrophy	Else et al. (129)

**Table 2:** Summary of genetic alterations in comprehensive studies.

Type of alteration	Gene		Chromosome	References
Gene mutation	*TP53*	Tumor protein p53	17p13.1	Zheng et al. (157)
	*CTNNB1*	Catenin beta 1	3p22.1	
	*MEN1*	Menin 1	11q13.1	
	*RPL22*	Ribosomal protein L22	1p36.31	
	*PRKAR1A*	Protein kinase cAMP-dependent type 1 regulatory subunit alpha	17q24.2	
	*NF1*	Neurofibromin 1	17q11.2	
Loss of heterozygosity	*ZNRF3*	Zinc and ring finger 3	22q12.1	
	*RB1*	RB transcriptional corepressor 1	13q14.2	
	*CDKN2A*	Cyclin-dependent kinase inhibitor 2A	9p21.3	
Increased copy number	*CDK4*	Cyclin-dependent kinase 4	12q14.1	
	*CCNE1*	Cyclin E1	19q12	
	*TERT*	Telomerase reverse transcriptase	5p15.33	
	*TERF2*	Telomeric repeat binding factor 2	16q22.1	
Altered expression	*MED12*	Mediator complex subunit 12	Xq13.1	Assie et al. (155)
	*DAXX*	Death domain-associated protein	6p21.32	
	*ZNRF3*	Zinc and ring finger 3	22q12.1	
Hypermethylation	*CDKN2A*	Cyclin-dependent kinase inhibitor 2A	9p21.3	Fonseca et al. (167)
	*GATA4*	GATA binding protein 4	8p23.1	
	*BCL2*	Apoptosis regulator	18q21.33	
	*DLEC1*	Deleted in lung/esophageal cancer 1	3p22.2	
	*HDAC10*	Histone deacetylase 10	22q13.33	
	*PYCARD*	PYD and CARD domain containing	16p11.2	
	*SCGB3A1*	Secretoglobin family 3A member 1	5q35.3	

**Table 3:** miRNA in ACC.

miRNA	Objective	References
miR-503, miR-1202, and miR-1275	Prognostic value	Ozata et al. (178)
miR-483, miR-195, and miR-497	Diagnostic value	Ozata et al. (178)
miR-483-5p	Diagnostic value	Patterson et al. (179)
		Patel et al. (175)
miR-195 and miR-100	Biomarker in ACCs	Patterson et al. (179)
miR-675 and miR-335	Diagnostic value	Schmitz et al. (180)
miR-195 and miR-483-5p	Prognostic value	Chabre et al. (174)
		Soon et al. (173)
MiR-34a	Diagnostic value	Patel et al. (175)
miR-503-5p, miR-483-3p, miR-450a-5p, miR210, miR-483-5p, miR-421	Diagnostic value	Koperski et al. (177)
